# Exon 21 deletion in the *OPHN1* gene in a family with syndromic X-linked intellectual disability: Case report

**DOI:** 10.1097/MD.0000000000021632

**Published:** 2020-08-14

**Authors:** Alina Bogliş, Adriana S. Cosma, Florin Tripon, Claudia Bãnescu

**Affiliations:** a Laboratory of Medical Genetics, Emergency Clinical County Hospital Târgu Mureş, Târgu Mureş¸ Romania; b Department of Genetics, George Emil Palade University of Medicine, Pharmacy, Sciences, and Technology of Târgu Mureş, Târgu Mureş, Romania; c Laboratory of Molecular Biology/Genetics, Center for Advanced Medical and Pharmaceutical Research, George Emil Palade University of Medicine, Pharmacy, Sciences, and Technology of Târgu Mureş, Târgu Mureş, Romania.

**Keywords:** brain anomalies, MLPA, *OPHN1* gene, strabismus, X-linked intellectual disability

## Abstract

**Introduction:**

The oligophrenin-1 (*OPHN1*) gene, localized on the X chromosome, is a Rho-GTPase activating protein that is related to syndromic X-linked intellectual disability (XLID). XLID, characterized by brain anomalies, namely cerebellar hypoplasia, specific facial features, and intellectual disability, is produced by different mutations in the *OPHN1* gene.

**Patient concerns:**

In this report, we present the clinical and molecular findings of a family affected by a mild XLID due to a deletion in the *OPHN1* gene, exon 21, Xq12 region using Multiplex Ligation-dependent Probe Amplification (MLPA) analysis. The clinical features present in the family are a mild developmental delay, behavioral disturbances, facial dysmorphism, pes planus, nystagmus, strabismus, epilepsy, and occipital arachnoid cyst.

**Interventions:**

The MLPA analysis was performed for investigation of the copy number variations within the X chromosome for the family.

**Diagnosis and outcome:**

The MLPA analysis detected a deletion in the *OPHN1* gene, exon 21 for the proband, and a heterozygous deletion for the probands mother. The deletion of the Xq12 region of maternal origin, including the exon 21 of the *OPHN1* gene, confirmed for the probands nephew.

**Lessons:**

Our findings emphasize the utility of the MLPA analysis to identify deletions in the *OPHN1* gene responsible for syndromic XLID. Therefore, we suggest that MLPA analysis should be performed as an alternative diagnostic test for all patients with a mild intellectual disability associated or not with behavioral disturbances, facial dysmorphism, and brain anomalies.

## 1 Introduction

Intellectual disability (ID), known as mental retardation (MR), is characterized by important limitations in intellectual functioning and also in adaptive behavior. The ability to find a solution for different problems, understand complex ideas, learn quickly, and learn from experience defined good intellectual functioning. Cognitive functioning can be measured by the intelligence quotient (IQ), which in patients affected with ID is equal to or below 70.^[1,2]^ The severity of ID ranges from moderate (IQ level 35–40 to 50–55) to severe (IQ level 20–25 to 35–40), with intrafamilial variability.^[2,3]^ All the skills learned and accomplished by people daily characterize adaptive behavior.^[1]^ ID represents a reduction in cognitive abilities that appear before the age of 18 years and influences approximately 1% to 3% of the general population.^[4,5]^

The etiology for ID is complex, including environmental factors as well as genetic defects, many of which signify X-linked intellectual disability (XLID).^[4–6]^ XLID refers to a group of hereditary disorders characterized by different degrees of intellectual disability produces by mutations in several genes present on the X chromosome.^[1]^ It is now known that intellectual disability is 30% to 40% more frequent in men than in women. One explanation for this may have to do with the existence of a single X chromosome in males (with normal karyotype: 46,XY). Therefore, all changes on the X chromosome will be manifested in boys.^[7]^ Mandel and Chelly have suggested that there may be some variations between gender, mainly in the fetal development of men when their brain is more susceptible to early damage.^[8]^

There are more than 100 XLID genes, and due to the remarkable percentage of non-syndromic XLID cases without any additional distinctive features, the diagnostic became a great challenge and the etiology of many cases remains unknown.^[1,6]^ One of them is the oligophrenin-1 gene (*OPHN1*), which is related to syndromic XLID, contains 25 exons, and is located in the Xq12 region containing 391Kb, encoding an 802 amino acid Rho GAPs domain.^[7]^*OPHN1* is a Rho-GTPase activating protein that is involved in the regulation of the G-protein cycle and is responsible for a syndromic form of XLID.^[3,9]^ This protein is observed both in glial cells and neurons where it colocalizes with F-actin, especially at the tip of rising dendrites and at both parts of the synapse.^[10]^ According to the Human Gene Mutation Database (www.hgmd.cf.ac.uk/ac/index.php) in subjects with ID have been reported 31 mutations publicly available consisting of 2 complex rearrangements, 8 missense/nonsense mutations, 4 small deletions and a small insertion, 11 gross deletions, 3 gross insertions/duplications, and 3 splicing mutations in the *OPHN1* gene. The recognizable phenotype includes intellectual disability, a distinctive facial appearance with strabismus, brain anomalies such as cerebral ventricular augmentation and cerebellar hypoplasia preponderant in the vermis, ataxia, seizures, and hypogenitalism.^[3,9]^*OPHN1* is omnipresent expressed in fetal but also in the adult brain, especially in the hippocampus, cerebellum, and olfactory bulb.^[3,10]^

Choosing the proper genetic test can be a challenge due to financial limitations in some parts of the world. A useful method in detection of genomic variation liable for ID patients is represented by multiplex ligation-dependent probe amplification (MLPA) analysis and array Comparative Genomic Hybridisation (array-CGH) with an average detection rate up to 20%.^[11,12]^ It was revealed that MLPA analysis represents a fast, easy and valuable method for identification of the copy number variations (CNVs), such as deletions and duplications, and to establish the origin of small supernumerary marker chromosome (sSMC) or to detect some point mutation; and this may represent a complementary alternative to array-based techniques for routine investigations.^[4,12,13]^ The entire genome may be screened for chromosomal gains and losses using array-CGH. This tool has some disadvantages because it cannot identify point mutations, balanced chromosomal rearrangements, losses, and gains in other parts of the genome, which are not covered by the array.^[14]^

Herein, we report clinical and molecular findings from a family affected by a mild XLID, due to a deletion in the *OPHN1* gene, exon 21, Xq12 region using MLPA analysis, and array-CGH analysis.

## 2 Case report

### 2.1 Subjects

**Figure 1:**
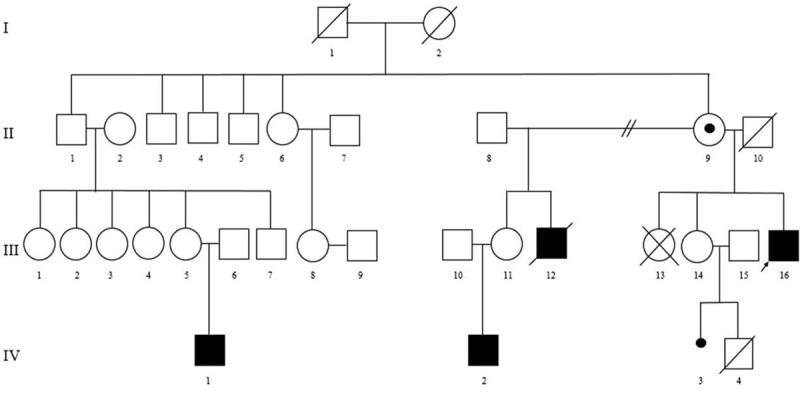
Family pedigree showing the segregation of the deletion in the *OPHN1* gene, exon 21, Xq12 region ascertained through proband III.16. Solid squares represent boys with ID. The circle with a black dot represents an unaffected carrier female.

The proband (III-16, Fig. 1), a 17-year-old boy, was referred to the Genetics Department of the Clinical Emergency County Hospital from Târgu Mureş, Romania, for the presence of a mild intellectual disability and behavioral disturbances involving aggressiveness. Taking into consideration the affected males in the family, after conducting the family history and assessing the family pedigree, we assumed an ID with an X-linked inheritance pattern. We performed MLPA analysis for the investigation of the CNVs within the X chromosome for the proband. Further investigations were conducted through MLPA analysis for the mother (II-9) and the sister (III-14) of the proband in our laboratory, and through array-CGH for the nephew (IV-2), in another laboratory.

## 3 Methods

For molecular analysis, fresh peripheral blood (2 ml) was collected into ethylenediaminetetraacetic acid (EDTA) vacutainer tube from the subjects (III-16, II-9, and III-14). Genomic DNA was isolated and purified from samples following the manufacturers instructions using the PureLink gDNA Blood Kit (Invitrogen, Carlsbad, CA, USA).

The MLPA analysis was performed by using the SALSA MLPA probemix P106-C1 MRX kit (MRC-Holland, Amsterdam, Netherlands). The P106-C1 MRX kit consists in 46 probes for detection of the CNVs of the 16 XLID genes [*RPS6KA3*, *ARX*, *IL1RAPL1*, *TSPAN7*, *PQBP1*, *HUWE1*, *OPHN1*, *ACSL4*, *PAK3*, *DCX*, *AGTR2*, *ARHGEF6*, *FMR1*, *AFF2* (*FMR2*), *SLC6A8* and *GDI1*] on the X chromosome which have been involved in (non-specific) X-linked intellectual disability (www.mlpa.com). The Coffalyser.Net software provided by the manufacturer was used to interpret the results.

The Ethics Committee from the George Emil Palade University of Medicine, Pharmacy, Science, and Technology of Târgu Mureş, Târgu Mureş, Romania, approved the protocol (66/27.04.2018), and informed consent for genetic testing and publication was obtained from the investigated subjects in accordance with the Declaration of Helsinki.

## 4 Results

### 4.1 Cases presentation

The proband (III-16) was the third child of non-consanguineous parents. He was born at term from normal pregnancy by vaginal delivery. At the age of 7 months, the proband presented convergent strabismus, congenital horizontal nystagmus, generalized muscular hypotonia, and reduced segmental force of the lower limbs. At the age of 11 months, he developed the first seizure and was suspected of craniosynostosis associated with mild psychomotor and language delay. The normal results on the skull X-rays excluded craniosynostosis. At the age of 13 years, electroencephalography (EEG) described epileptiform modifications.

The genetic examination of the proband (III-16) revealed: facial dysmorphism with a prominent forehead, a long face, ocular anomalies (bilateral ptosis, horizontal nystagmus, convergent strabismus of the right eye and impaired visual perception, deep-set eyes), prominent nose with wide nasal base, high palate, dental cavities, prognathism, and thoracic kyphoscoliosis, pes planus, and long fingers.

At the neurological examination, the proband showed poor fine motor coordination and the inability to organize instrumental actions.

The psychological examination included: a mild coordination deficit with a low spatial orientation, mechanical memory with short term memory capacity, emotional instability, and poor concentration. The proband intelligence quotient (IQ) is estimated to be 67, corresponding to mild intellectual disability. During childhood, he had poor interaction with other children, psychomotor agitation, language delay, poor vocabulary, and dyslexia.

The echocardiography showed a patent foramen ovale and a stage 3 tricuspid insufficiency. The brain-computer tomography (CT) revealed a left occipital arachnoid cyst and a left maxillary mucocele of 14 mm.

The ophthalmological assessment revealed horizontal nystagmus, hypermetropic astigmatism, convergent strabismus of the right eye, and impaired visual perception, amblyopia. The ophthalmoscopy was normal.

The second case, the subject IV-2, is a 5-year-old boy from a full-term pregnancy by vaginal delivery complicated by imminent abortion. Developmental milestones include head control at 4th months, sitting position after 7th months, and walking at the age of 24th months. At the last clinical examination were observed the following characteristics: discrete facial dysmorphism, the presence of lenses for convergent bilateral strabismus, single palm crease, pes planus, and genu valgus. The neurological examination revealed a watchful patient with an intact state of consciousness, indifferent attitude with motility and walking, and uncontrolled sphincters. Psychomotor development showed an overall psychomotor development below the expected motor age. The personal and social scale was equivalent to a child aged 34.5 months, a performance scale of 28 months, and language and understanding scale of 19 months. The imagistic investigation included normal results for abdominal ultrasounds. The electrocardiogram (ECG) was normal.

### 4.2 Genetic analysis

**Figure 2:**
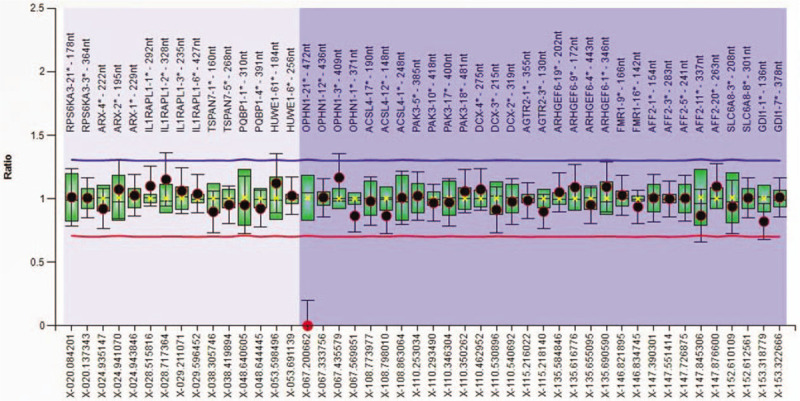
Ratio chart for MLPA analysis using SALSA MLPA probemix P106-C1 MRX in the proband (III.16).

**Figure 3:**
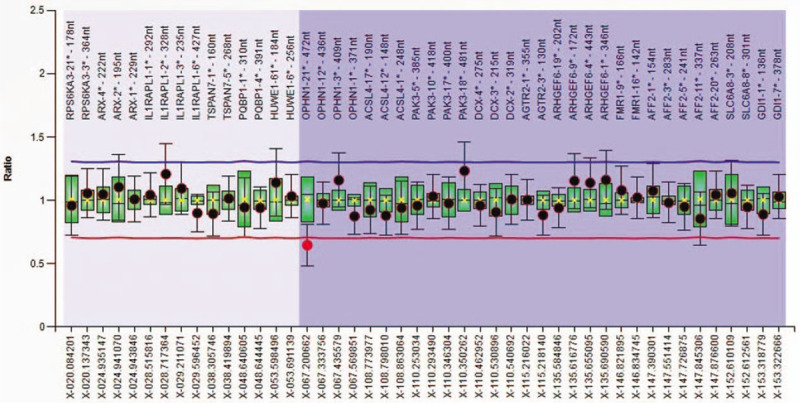
Ratio chart for MLPA analysis using SALSA MLPA probemix P106-C1 MRX in the probands mother (II.9).

The MLPA analysis was carried out for the proband (III-16), probands mother (II-9) and for the sister (III-14). The MLPA analysis identified the absence of signal for the *OPHN1* probe, at the site of the exon 21, in the *OPHN1* gene, on the level of the Xq12 region (Fig. 2), interpreted as a deletion of the *OPHN1* gene. The MLPA analysis of the probands mother (II-9) detected a reduced signal of the same probe for the *OPHN1* gene (the dosage quotient of the probe <0.65), interpreted as a heterozygous deletion in exon 21 of the *OPHN1* gene (Fig. 3). In probands sister (III-14) probes have not suggested any changes. Our molecular findings are recommended to be confirmed by another molecular technique, according to the MRC-Holland (www.mlpa.com).

In parallel, the probands nephew (IV-2) was investigated in another laboratory for the confirmation of our results. The array-CGH analysis by using CytoSure needle ISCA V2.0 8X60K OGT (Oxford Gene Technology, United Kingdom) identified a microdeletion of the long arm of the Xq12 region extended about 16 Kb, of maternal origin with the array-CGH formula according to the International System for Human Cytogenetic Nomenclature (ISCN) 2013 arr[hg19] Xq12(67,283,190-67,299,198)x1 mat. The deletion identified through MLPA analysis in a family with mild ID was confirmed by array-CGH analysis.

## 5 Discussion

Nowadays, the *OPHN1* gene is considered to be implicated in syndromic XLID with facial dysmorphism and brain anomalies.^[15]^ Also, these patients revealed similar clinical and radiologic characteristics, particularly in the cerebellum and ventricles.^[16]^

In the present study, we report the clinical and molecular aspects of a family with a characteristic phenotype for *OPHN1* syndrome, including mild ID, brain anomaly, and facial dysmorphism with a prominent forehead, long face, nystagmus, convergent strabismus, deep-set eyes, prognathism, and a prominent nose. In this family, the phenotype of the 2 mild affected boys was suggestive of XLID. A probands male cousin (IV-1) was clinically diagnosed with a mild intellectual disability, but no further investigations have been done. Probands nephew presents (IV-2) discrete facial dysmorphism, convergent bilateral strabismus, single palmar crease, pes planus, and genu valgus. To date, the mutations detected in the *OPHN1* gene have been described in subjects with neurodevelopmental diseases such as ID, ataxia, epilepsy, seizure, and schizophrenia.^[17]^

The facial features associated with *OPHN1* syndrome reported by Ronzoni et al and Al-Owain et al comprise prominent supraorbital ridges, deep-set eyes, hypotelorism, thin upper lip, prognathism, and a short philtrum.^[9,18]^ Only subject III-16, in our family report, presents the prognathism and deep-set eyes. The cerebellum is involved in oculomotor coordination, which may contribute to nystagmus and strabismus. The oculomotor problems which seem to appear due to hypoplasia of the cerebellum many times remarked on brain imaging studies.^[19]^ Strabismus represents a sign found in approximately 90% of previous reported *OPHN1* mutations and is present in both investigated children.^[3,8,9,15,16,18,20–23]^ Nystagmus has been described by Moortgart et al,Schwartz et al, and Zanni et al,^[8,21,24]^ and it is also present in the proband (III-16). The facial dysmorphism seen in almost all affected patients could be explained by the expression of *OPHN1* in the craniofacial bones.^[24]^ Tall stature and macrocephaly have also been described in different families,^[19]^ but not present in our investigated subjects (III-16, IV-2).

Several authors reported cases with cryptorchidism, micropenis, hypoplastic scrotum connected with the *OPHN1* gene,^[3,22,24]^ but in our study, none of our subjects presents abnormal external genitalia.

Intellectual disability, speech delay, and behavioral disorder are highlighted in several reports in patients with *OPHN1* gene mutations.^[3,8,18,21–25]^ As a similarity in this study, the proband (III-16) investigated in our laboratory presents these characteristics. For now, we do not have enough clinical data and genetic investigations performed in the other family members except probands mother (II-9), sister (III-14), and nephew (IV-2). The lack of compliance and the precarious financial situation of the family have led to the impossibility of carrying out more investigations.

The most common Magnetic resonance imaging (MRI) brain scan anomalies reported in different studies were cerebellar hypoplasia, especially of the lower vermis, enlarged cisterna magna, or retrocerebellar cysts, the anterior vermis may be disorganized and also hippocampal alteration.^[4,9,20]^ MRI brain scans in the subject IV-2 denoted a slight reduction in the volume of the lower cerebellar vermis, large lateral ventricles, which is associated with an increase in the width of the corresponding liquor spaces, a cerebral malformation compatible with Dandy-Walker variant. The EEG recordings showed mild anomalies of cerebral electrical activity in the frontal area bilaterally. Our proband presented an occipital arachnoid cyst without any other modifications.

Because of the phenotypic variability of the XLID, carries are frequently reported as asymptomatic with a non-specific and mild phenotype (strabismus and learning difficulties). Therefore, this theory is consolidated by the inactivation of the X chromosome with *OPHN1* mutations.^[3,19]^ The probands mother (II-9) has a borderline intelligence and present a heterozygous deletion in exon 21 of the *OPHN1* gene. The sister (III-14) also has a borderline intelligence and developed epilepsy from the age of 3 years old. Al-Owain et al described a case of a girl with a mild form of cerebellar hypoplasia diagnosed following an MRI scan.^[18]^ Menten et al reported a girl with neuroradiological abnormalities (vermian and brain hypoplasia and enlarged the fourth ventricle),^[19]^ Bergmann et al reported a woman with normal MRI scans.^[23]^ None of the women in our study have been neuroradiologically investigated due to a lack of compliance and geographical dispersion of the family members.

For the confirmation of our MLPA analysis results in the family, the array-CGH was performed for the subject IV-2 in other laboratory and revealed a change in the number of copies, a microdeletion of the long arm of the X chromosome involving the Xq12 region extended about to 16 Kb, of maternal origin; microdeletion that also include exon 21 of the *OPHN1* gene.

## 6 Conclusion

Diagnosing patients with X-linked intellectual disability remains a difficult task among clinicians. Following the genetic examination, the diagnosis represents a challenge, and an enormous benefit is brought by applying the latest available molecular tests. We underline the value of MLPA analysis, a liable, practical and complementary alternative to next-generation sequencing, whole-genome or whole-exome sequencing, in the identification of deletions in the *OPHN1* gene in patients with mild ID. MLPA is recommended as an alternative diagnostic test for the patients with a mild ID associated or not with behavioral disturbances, facial dysmorphism, and brain anomalies. Further genetic investigations are needed to detect the carriers in other members of the family and to assess the recurrence risk for future pregnancies.

## Author contributions

**Conceptualization:** Claudia Bãnescu.

**Data curation:** Alina Boglis, Florin Tripon, Claudia Bãnescu.

**Formal analysis:** Alina Boglis, Florin Tripon, Claudia Bãnescu.

**Investigation:** Alina Boglis, Adriana S. Cosma, Florin Tripon.

**Methodology:** Claudia Bãnescu.

**Supervision:** Claudia Bãnescu.

**Writing – original draft:** Adriana S. Cosma.

**Writing – review & editing:** Alina Boglis, Florin Tripon, Claudia Bãnescu.
